# And therefore I have sailed the seas and come: To the holy city of Byzantium

**DOI:** 10.3201/eid1705.AC1705

**Published:** 2011-05

**Authors:** Polyxeni Potter

**Affiliations:** Author affilation: Centers for Disease Control and Prevention, Atlanta, Georgia, USA

**Keywords:** art science connection, emerging infectious diseases, art and medicine, Stelios Faitakis, Kakerlaken sind die Zukunft, And therefore I have sailed the seas and come, To the holy city of Byzantium, Byzantine tradition, vector-borne infections, Sailing to Byzantium, W.B. Yeats, about the cover

―William Butler Yeats

“One morning, as Gregor Samsa was waking up from anxious dreams, he discovered that in his bed he had been changed into a monstrous verminous bug,” wrote Franz Kafka in The Metamorphosis. “He lay on his armor-hard back and saw, as he lifted his head up a little, his brown, arched abdomen divided up into rigid bow-like sections. From this height the blanket, just about ready to slide off completely, could hardly stay in place. His numerous legs, pitifully thin in comparison to the rest of his circumference, flickered helplessly before his eyes.”

Kafka’s nightmarish tale captures the essence of unexpected uncontrollable life-defining horror, likely caused by Gregor Samsa’s inability to cope with societal and family pressures. His predicament, much like any severe disfiguring and disabling illness, would isolate and eventually kill him. Transformative experiences have been the domain of poets and artists alike because artistic sensibility heightens awareness of reality, prompting them to seek a better or more comprehensible alternative. Such seems to be the goal of Stelios Faitakis, who from his native Greece has set out to understand and convey to all a meaningful version of the world around him.

“I was raised in the west suburbs of Athens,” recounted Faitakis in a 2007 interview, “a place occupied mostly by the working class.” A serious injury during childbirth left him with substantial paralysis of the right arm and, despite extensive surgical and other interventions, it would limit his activities, including painting, to his left arm. “Both my parents were workers in a gold chain factory…. They are not what we’d call ‘artists,’ although when I see my mother creating new designs… my artistic nature has some root there…. Also my grandfather… was a good draftsman…. I have painted since childhood.”

Overcoming family opposition, Faitakis found his way into the prestigious Academy of Fine Arts in Athens, where he set out on his artistic journey, guided as much by his affinity to mathematics as by Eastern mysticism. Suspicious of authority and large organized institutions, he is self-reliant and openly critical of political oppression. “As long as I can remember, I’ve been an anarchist.” He views art as an inclusive and enlightening agent. His first efforts to reach the public came as graffiti―in the streets of his hometown and later on the walls of the Academy. “Working for the public for me means mostly painting outdoors in the open, in the streets, where everyday people pass to go to their jobs…. Athens… begs to be painted.” His murals have now dotted the globe, from European cities to Miami’s Wynwood Art District.

“From the beginning, I chose to paint narrative pictures, like a still from a theatrical play: human characters in some environment doing some action—the simplest scenario possible,” with hidden meanings, “as an extra for the more demanding eyes.” His heavily populated canvases and murals of common folk at work and play have been likened to the paintings of Pieter Bruegel the Elder; Mexican muralism, a monumental form of wall painting accessible to the masses; Japanese screen painting; and the Cretan School, whose style of icon painting, also seen in the work of El Greco, flourished during the late Middle Ages and peaked after the fall of Constantinople, becoming a major force in Greek painting during the 15th through the 17th centuries.

But what has brought Faitakis into his own is a consistent reference to art forms rooted in the Byzantine tradition. “It would never be possible for me to neglect this element…. I paint about Humanity and its relation to itself… so my characters flow in a golden world…. Simple, ordinary colors coexist with metallic/light reflecting colors…. The gold refers to eternity, universal time.” The tradition relies on exegesis, “Art should be used as a tool for human beings to… grow.” In Faitakis’ work, monastic settings, precipitous mountains, and hermetic deserts unite with urban scenes, political unrest, and common ailments to seek resolution in unorthodox ways. “Art opens the human being to the use of capabilities that our modern civilization has shrunk, such as intuition and inspiration.”

In *Kakerlaken sind die Zukunft*, on this month’s cover, repetitive detailed patterns and elements from psychology, the natural sciences, popular wisdom, and political allegory unite with humor and drama to create aggressive commentary. Perspective is achieved by stacking objects on top of each other. Buildings, decaying cities and surroundings, complex geometric and floral designs, and message ribbons portray, without physical barriers or reference to place and time, the universe. Size denotes importance, so the insect containing the urban scene, placed in full frontal position and regalia, spells an ominous message: vermin can outnumber, outdo, and outlive the human community. Larger than life and wearing a person’s head, this all-weather vector feeds on poverty, ignorance, disease, and death.

Frustrated by the state of affairs, the painter abandons the mundane and, like W.B. Yeats, sails to Byzantium—not a destination but an idea. For as the poet says, “the only way for the soul to learn to sing is to study “monuments of its own magnificence.” In the vernacular of a bygone culture, he transcends reality to transform obscure troublesome prospects to legible content accessible to all. As the Kafkaesque scenario unfolds, modern inventions move in and out the stylized landscape―a giant electronic screen; helicopters buzzing like giant insects with petal-like rotors, steam haloes, and landing limbs. A tiny human lurks in a shadowy crevice on the side. Beyond exegesis, this eerie scene invites anagogical interpretation.

Much like a scientist with a microscope, Faitakis amplifies figures and their surroundings to take a closer look at vermin and force the viewer to experience their presence and resilience. But in painting this anthropomorphic insect, he also creates an icon of the ubiquitous vector, which transmits viruses, bacteria, and other pathogens between humans and animals, forming a bridge over spatial, behavioral, and ecologic barriers and promoting the emergence of disease. Yellow fever virus is transmitted from monkeys to humans by mosquitoes; Lyme disease, from rodents to humans by ticks. And though Faitakis’ insect contains the city, a vector is not just a vessel. Always evolving as part of the elaborate transmission mechanism, a vector provides the pathogen itself ways to evolve, creating even more opportunities for disease emergence.

The complexity of human-animal-vector interactions underlying *Kakerlaken sind die Zukunft* adds another dimension to Faitakis’ surreal universe. And like the sociopolitical elements, these interactions are staggering and require deeper understanding that may reside “as in the gold mosaic of a wall” at the convergence of human behavior, vector biology, climate, and land use.

**Figure:**
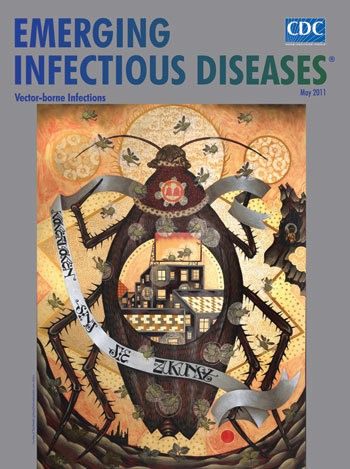
Stelios Faitakis (b. 1976) *Kakerlaken sind die Zukunft* (2009) Mixed media on canvas (260 cm × 190 cm). Courtesy of The Breeder gallery@thebreedersystem.com, Athens. Photo: Vivianna Athanasopoulou
